# Time‐Efficient, Accurate, and Experimentally Grounded Optical Modeling of Multiscale‐Textured Thin‐Film Solar Cells

**DOI:** 10.1002/gch2.202500448

**Published:** 2025-12-17

**Authors:** Federica Saitta, Govind Padmakumar, Paula Perez Rodriguez, Paul Procel Moya, Rudi Santbergen, Arno H.M. Smets

**Affiliations:** ^1^ Photovoltaic Materials and Devices (PVMD) Group Delft University of Technology Delft The Netherlands

**Keywords:** light management, multiscale‐textured interfaces, next‐generation photovoltaics, optical modeling, thin‐film solar cells

## Abstract

Accurate prediction of optical performance in solar cells with multiscale‐textured interfaces is essential for optimizing light management in next‐generation photovoltaics. For the first time, a systematic validation of two complementary modeling approaches is carried out on experimentally fabricated thin‐film silicon (TF Si) solar cells: rigorous coupled‐wave analysis (RCWA), offering a full electromagnetic solution but constrained by boundary conditions, and a ray optics model, operating in the refractive regime. The study involves two device architectures: an a‐Si:H single‐junction cell on commercial Asahi VU‐type glass with random nanotextures, and an nc‐Si:H single‐junction cell on novel micro‐periodic honeycomb‐textured glass developed in‐house. Simulated and measured external quantum efficiency (EQE) and total front reflection losses (1‐R) are benchmarked using the root mean squared error (RMSE). The ray model shows deviations of only 2%–6%, comparable to RCWA, while reducing computation time from 1 week to less than 30 min. Applied to an a‐Si:H/nc‐Si:H tandem device on honeycomb‐textured glass, ray optics reproduced the optical response with spectral deviations below 6% and photocurrent mismatch under 0.2 mA/cm^2^. These findings uniquely establish ray optics, when combined with accurate optical constants and realistic interface morphologies, as a reliable and computationally efficient predictive tool broadly transferable to thin‐film technologies, including perovskites.

## Graphical abstract

A time‐efficient and accurate optical modeling framework combines ray tracing with measured refractive index (n) and extinction coefficient (k) data, together with atomic force microscopy (AFM) surface profiles, to capture multiscale textures in thin‐film solar cells. The method reproduces reflection losses and predicts optical device performance across single‐junction and tandem architectures, and is readily applicable to next‐generation photovoltaic devices.

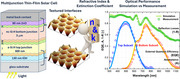

## 1 Introduction

Thin‐film (TF) Si‐based solar cells remain attractive for cost‐effective, lightweight, and scalable photovoltaic technologies [1, 2]. However, their relatively low optical absorption, particularly in the near‐infrared region of the solar spectrum, fundamentally limits device efficiency [3]. Effective light‐management strategies are therefore essential to minimize reflection losses and maximize photon absorption in both single and multijunction TF architectures [4, 5].

Previous approaches have employed engineered textured substrates, such as modulated surface textured (MST) electrodes [6], random and periodic Si textures [7], Asahi glass [8], and flexible laminates [9], with record efficiencies demonstrated using honeycomb patterns on crystalline Si wafers [10, 11]. Despite these advances, a comprehensive optical modeling capable of accurately predicting device performance and guiding the design of next‐generation textured solar cells is still lacking.

A fully integrated, experimentally validated optical model that simultaneously accounts for external quantum efficiency (EQE) and front reflection losses (1 − R) across realistic multiscale architectures has not yet been established [12, 13]. Prior studies have typically considered either EQE or reflectance in isolation, often under simplified geometries, leading to discrepancies between simulations and actual device performance [14, 15]. In this work, these metrics are unified within a single validated optical model, enabling accurate and quantitative assessment of the optical response and associated losses in complex textured thin‐film silicon solar cells.

Advancing optical modeling requires overcoming two key challenges. First, accurate prediction depends not only on the availability of advanced textured substrates but also on reliable characterization of the optical constants (refractive index and extinction coefficient) of all constituent materials. Second, realistic implementation of interface texturing is essential, as it strongly governs light scattering, trapping, and interference within the multilayer stack [16].

To address these challenges, two optical modeling approaches are investigated: rigorous coupled‐wave analysis (RCWA) [17] and ray optics [18]. The comparison is carried out using two textured glasses: the widely used but less optically characterized Asahi VU‐type glass with random nanostructures [19], serving as the substrate for a single‐junction hydrogenated amorphous silicon (a‐Si:H) solar cell, and a novel micro‐periodic honeycomb‐textured glass developed in‐house [20], used as the substrate for a single‐junction hydrogenated nanocrystalline silicon (nc‐Si:H) solar cell.

Rigorous coupled‐wave analysis provides a full‐wave solution capable of capturing complex interference and scattering effects by solving Maxwell's equations and is therefore expected to deliver high accuracy for fine‐scale textures. However, its application to complex, non‐periodic, or conformal structures is computationally demanding and requires extensive optimization [21, 22]. In contrast, the raytracing optical model offers significantly reduced computational effort but is often considered less reliable for nanoscale features [23].

In this study, RCWA and ray optics are systematically compared for the first time on experimentally fabricated a‐Si:H and nc‐Si:H single‐junction devices on commercial and in‐house–developed textured substrates, with the analysis further extended to a‐Si:H/nc‐Si:H tandem solar cell architecture. By integrating realistic interface morphologies, accurately measured optical constants, and validation against both EQE and reflection measurements, this work establishes the first experimentally validated optical modeling that simultaneously reproduces absorption and reflection in multiscale‐textured thin‐film devices, while balancing accuracy with computational efficiency. Beyond the specific cases investigated, this methodology provides a broadly applicable tool for predictive optical modeling of next‐generation textured photovoltaic technologies.

## 2 Optical Modeling Framework

The optical response of the solar cell structures is simulated using the GenPro4 solver [24], which employs the net‐radiation method with angular intensity distributions of reflection and transmission as input. This approach captures multiple reflections, transmissions, and scattering events, enabling realistic modelling of multilayer stacks with textured interfaces.

In GenPro4, each material within the solar cell stack is classified as either a layer or a coating, depending on how interference is expected to influence light propagation. This distinction is based on the optical thickness of the film relative to the coherence length of sunlight and on the optical contrast between adjacent layers. The coherence length defines the distance over which the phase of the electromagnetic wave remains correlated.

When this correlation is maintained, typically in thin or weakly absorbing films, reflected and transmitted waves can interfere, and the film is considered optically coherent. Such layers are modeled as coatings, where light propagation is treated in terms of field amplitudes including both magnitude and phase.

When the optical thickness is sufficiently large or absorption is strong, the phase correlation between multiple reflections is lost, and interference effects average out. These layers are considered optically incoherent and are modeled as layers, where only the light intensities are summed. This hybrid formalism enables GenPro4 to describe both coherent and incoherent optical behavior within the same simulation framework [25].

Another key input to GenPro4 is the complex refractive index of each material, defined by its refractive index (n) and extinction coefficient (k). These wavelength‐dependent optical constants determine the reflectance, transmittance, and absorptance within the stack. The implied photocurrent density (J_ph_) is calculated from the absorptance spectrum according to Equation (1) [26]:

(1) $$J_{\mathrm{ph}}=e\int_{\lambda_{1}}^{\lambda_{2}}A_{\mathrm{abs}}\left(\lambda \right)\cdot \Phi_{\mathrm{AM}1.5G}\left(\lambda \right)d\lambda$$

where 𝐴_abs_(λ) is the spectral absorptance of a single layer, Φ_AM1.5_ (λ) is the AM1.5G photon flux spectrum, and e is the elementary charge. The integration limits 𝜆_1_ and λ_2_ correspond to the lower and upper bounds of the spectral range, respectively, with 𝜆_2_ typically set by the optical bandgap of the absorber material.

The standard GenPro4 solver models light interaction with textured surfaces using two complementary approaches: ray optics and wave optics. The wave‐optics formalism is based on the scalar scattering theory developed by K. Jäger, which performs well for small‐scale textures such as Asahi U‐type glass but loses accuracy for larger or more complex morphologies [27]. Full‐wave Maxwell solvers can rigorously model light scattering but are computationally demanding and generally constrained to periodic boundary conditions. As a result, simulations are often restricted to small unit cells that do not represent the full complexity of random thin‐film morphologies. Rigorous coupled‐wave analysis offers a practical alternative, enabling accurate treatment of non‐periodic and sub‐wavelength textures at reduced computational cost. In this work, RCWA is integrated into GenPro4 as an alternative Maxwell solver.

For the validation study, ray optics and RCWA are applied independently within GenPro4, and their predictions are directly compared with the measured optical response of the fabricated solar cells. This strategy makes it possible to evaluate which model best reproduces the experimental EQE and reflection spectra while quantifying the trade‐off between computational efficiency and predictive accuracy.

The deviation between simulated and experimental spectra is quantified by calculating the root mean squared error (RMSE), expressed in percentage form as in Equation 2 [28]:

(2) $$\mathrm{RMSE}\left(\%\right)=\sqrt{\frac{1}{N}\sum_{i=1}^{N}{\left(X_{\mathrm{model}}\left(\lambda_{i}\right)-X_{\mathrm{meas}}\left(\lambda_{i}\right)\right)}^{2}}\;x\;100$$

where X_model_(λ_i_) and X_meas_(λ_i_) denote the simulated and experimental values at wavelength λ_i_ and, N is the number of spectral points. In this framework, X_model_ can represent either the simulated absorptance or the simulated total front reflection losses, while X_meas_ corresponds to the external quantum efficiency or the measured total front reflection losses, respectively. When simulated and experimental spectra are sampled on different wavelength grids, the simulated data are linearly interpolated onto the experimental grid prior to error evaluation.

### 2.1 Rigorous Coupled‐Wave Analysis

In RCWA, the model simplifies the problem by slicing the solar cell into thin sublayers along the propagation direction (z‐axis), while allowing arbitrary structural variations in the transverse plane (x–y directions). Maxwell's equations are solved using a semi‐analytical approach in the Fourier domain: analytically along the z‐axis and numerically in the x and y directions. In theory, an infinite number of Fourier harmonics would be required to fully resolve any arbitrary geometry, but in practice, the expansion must be truncated. A convergence analysis performed in this study shows that 17 Fourier modes are sufficient to obtain stable reflectance and absorption spectra for the investigated thin‐film Si structures. Higher orders introduce only marginal differences while significantly increasing computation time. The optimal number of modes is case‐specific and depends on factors such as texture height, feature shape, and the refractive index contrast within the structure. Therefore, a preliminary convergence analysis is essential to determine the minimum number of Fourier modes that capture the relevant physical behavior without incurring unnecessary computational overhead.

In addition to harmonic truncation, the spatial discretization of morphology must be sufficiently fine to resolve the local electric field distribution ($\vec{E}$) in x, y, and z directions. As with Fourier modes, the grid resolution requires balancing accuracy with computational feasibility, since finer discretization improves field representation but significantly increases simulation time.

The RCWA simulations are executed on a workstation equipped with an AMD EPYC 7552 48‐core processor and 1024 GB RAM. A complete wavelength sweep for each solar cell configuration requires approximately 1 week of computation time.

#### 2.1.1 Tukey Window Function

Since RCWA relies on periodic boundary conditions, accurate modelling of experimentally measured morphologies requires preprocessing of atomic force microscopy (AFM) height maps. While the ray‐tracing model uses the AFM matrix directly, RCWA needs a smoothed input to suppress edge artifacts at the unit cell boundaries.

**FIGURE 1:**
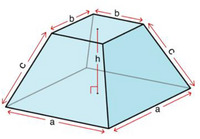
Geometric representation of the 3D Tukey window function used to smooth AFM height maps prior to RCWA simulations. Parameters are the base length a, the top length b, the frustum height h, and the lateral face length c.

To address this, the AFM height matrices are processed using a 2D Tukey window function [26]. The window gradually tapers height values at the edges of the domain, reducing discontinuities while preserving central features of the measured profile. The smoothing is controlled by an overall lateral rescale factor r = 0.2, which provides sufficient tapering at the edges without significant loss of morphological detail. In this case, the central 64% of the AFM map remains completely unchanged, while the surrounding 36% is gradually suppressed, ensuring that the core morphology is preserved while unit‐cell discontinuities are minimized. Smaller values of r leave sharp edges, whereas larger values overly suppress the central topography. Geometrically, smoothed morphology resembles a frustum of a square pyramid with isosceles trapezoidal lateral faces, as illustrated in Figure 1.

The frustum height is set to h = 1, the side length of the base equals the AFM pixel dimension a, and the top face length is b = (1−r)·a. The lateral face length c is calculated in Equation 3 as:

(3) $$c=\sqrt{h^{2}+{\left(\frac{ra}{2}\right)}^{2}}$$

## 3 Simulation Input

This section outlines the simulation inputs required to predict the optical performance of the solar cell structures. Emphasis is placed on the accurate representation of textured interfaces and material optical properties, both of which are critical for obtaining realistic results with ray optics and RCWA. Section 3.1 details the morphological characterization of the textured glass substrates. Sections 3.2 and 3.3 describe the device architectures and layer stacks of the a‐Si:H and nc‐Si:H single‐junction solar cells, along with the corresponding refractive index and extinction coefficient spectra of the constituent layers. Finally, Section 3.4 summarizes the key differences between the two optical models in tabular form.

### 3.1 Morphological Characterization of Textured Glass Substrates

A widely used substrate for TF Si‐based solar devices is the commercial SnO_2_:F (FTO)‐coated glass from Asahi Glass Company [29, 30]. The earlier Asahi U‐type glass featured random pyramidal facets with moderate slopes, designed to enhance light scattering while minimizing potential electrical losses [31]. The shift from U‐type to the current VU‐type substrates was motivated by the need to enhance long‐wavelength light scattering (600–1000 nm), crucial for improving the performance in multijunction solar cells [32, 33]. While the U‐type has been extensively characterized, detailed optical data on VU‐type glass remains limited. The increasing use of VU‐type substrates in high‐performance TF Si devices, including this work, underscores the importance of accurate optical and morphological characterization for reliable modeling and device design.

**FIGURE 2:**
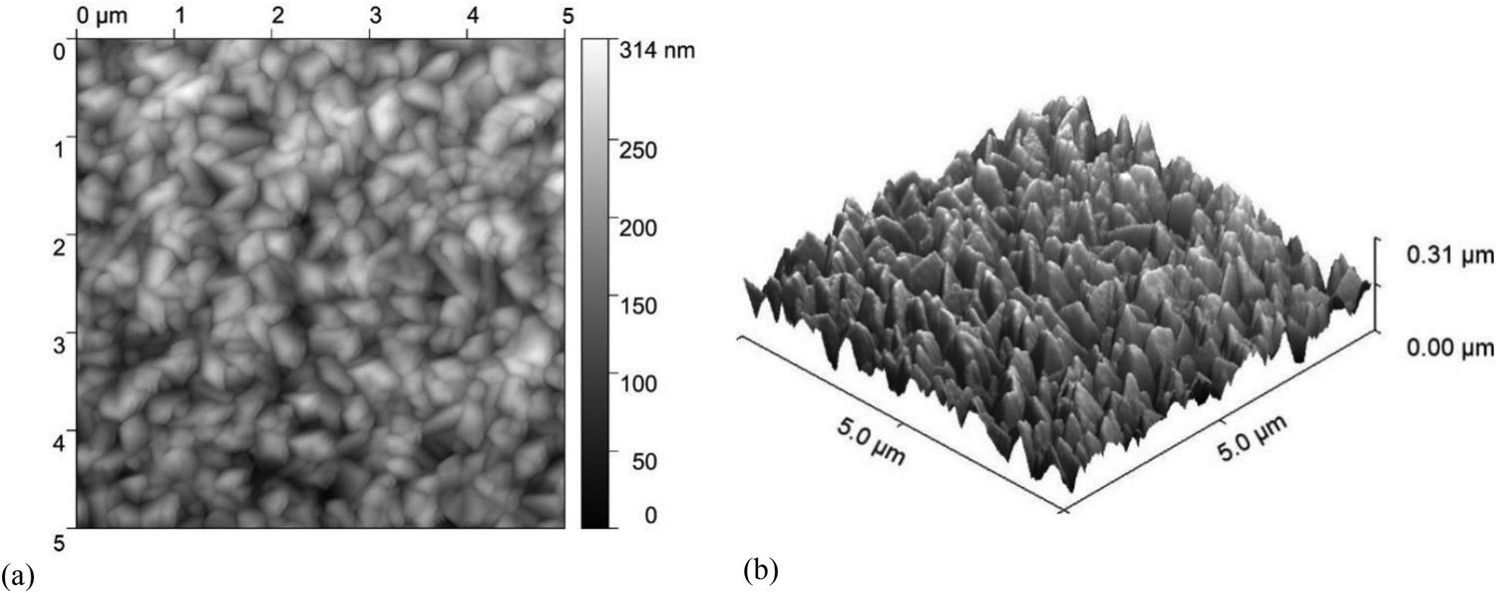
Atomic force microscopy (AFM) images of the Asahi VU‐type glass substrate: (a) 2D top‐view and (b) 3D morphology. The random texture exhibits a root mean square roughness (σ_RMS_) of 45 nm over a 5 µm × 5 µm area.

Figure 2 shows the AFM characterization of the Asahi VU‐type glass surface. The morphology exhibits a complex random nanoscale texture with peak‐to‐valley heights of approximately 0.31 µm over a 5 µm × 5 µm area, with a root mean square roughness (σ_RMS_) of 45 nm.

Random textures, such as those of Asahi glass, scatter light diffusely without selectivity, which limits their ability to redirect light into oblique guided paths or efficiently scatter at longer wavelengths. This reduces their effectiveness in devices such as nc‐Si:H solar cells, where absorption extends beyond 1 µm. In contrast, engineered periodic textures enable controlled light scattering, diffraction, and guided‐mode coupling, improving absorption across a broader spectral range.

**FIGURE 3:**
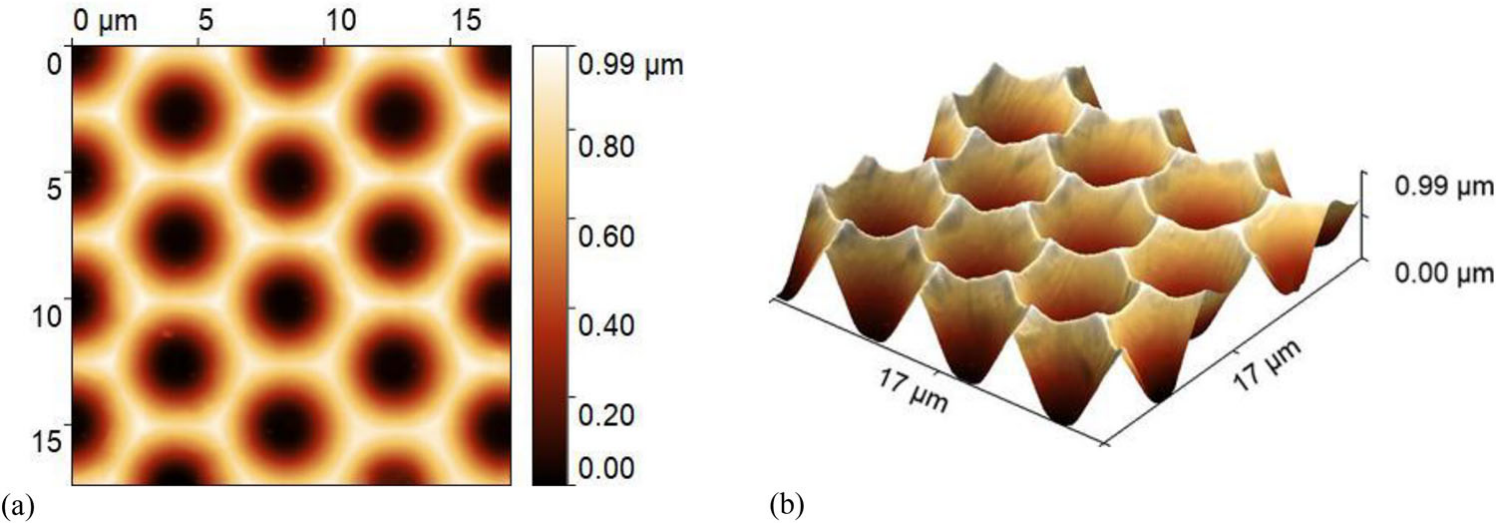
Atomic force microscopy (AFM) images of the honeycomb‐textured glass substrate: (a) 2D top‐view and (b) 3D morphology. The periodic structure exhibits a root mean square roughness (σ_RMS_) of 262 nm over a 17 µm × 17 µm area.

Figure 3 displays the AFM characterization of the in‐house fabricated honeycomb‐textured glass substrate. The morphology exhibits a highly uniform and periodic microstructure with σ_RMS_ of 262 nm. The well‐defined geometry enables precise angular redistribution of light into the absorber, enhancing light trapping, particularly in thick nc‐Si:H devices that require a strong long‐wavelength response.

### 3.2 a‐Si:H Single‐Junction Solar Cell on Asahi VU‐Type Substrate

**FIGURE 4:**
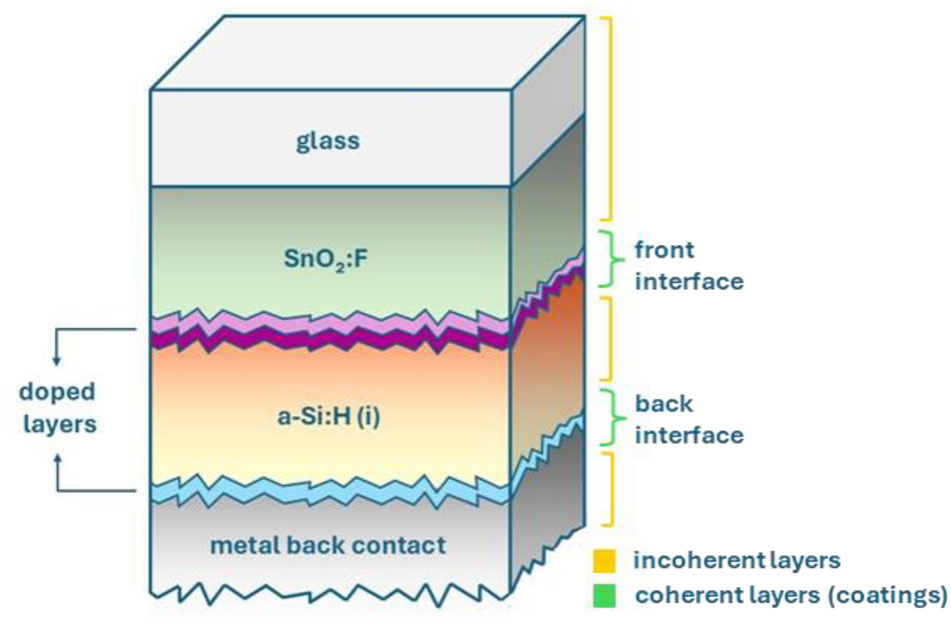
Schematic cross‐section of the a‐Si:H single‐junction solar cell on Asahi VU‐type glass (not to scale). Layer sequence: Asahi VU‐type glass (1.1 mm) with SnO_2_:F (700 nm)/AZO (10 nm)/p‐type nc‐SiO_x_ (10 nm)/intrinsic a‐SiO_x_ (3 nm)/intrinsic a‐Si:H (300 nm)/n‐type nc‐SiO_x_ (20 nm)/Ag back reflector (300 nm).

On the Asahi VU‐type substrate, a p‑i‑n a‑Si:H single‐junction solar cell is fabricated in a superstrate configuration. This architecture exploits the enhanced light scattering of VU‐type glass to improve optical confinement and device performance. The solar cell structure is illustrated in Figure 4 (not to scale).

The device is deposited on a 1.1 mm Asahi VU‐type glass coated with a 700 nm SnO_2_:F layer, which functions as the transparent front electrode. The intrinsic a‐Si:H absorber layer is 300 nm thick, with p‐type and n‐type regions of 10 to 20 nanometers located at the front and rear, respectively. Both the p‐type and n‐type layers are composed of nanocrystalline silicon oxide (nc‐SiO_x_). A 10 nm thick aluminum‐doped zinc oxide (AZO) buffer layer is inserted between the substrate and the p‐type layer. In addition, a 3 nm intrinsic a‐SiO_x_ layer is added between the p‐type nc‐SiO_x_ and the intrinsic absorber layer. This thin oxide effectively blocks boron diffusion into the absorber, eliminating the need for additional processing steps [34]. The rear contact consists of a 300 nm silver reflector. Experimentally, the back electrode stack includes silver (Ag), chromium (Cr), and aluminum (Al). However, since Cr and Al do not influence the optical performance, they are excluded from the optical design of the solar cell.

All subsequent layers conform to the textured substrate, so each interface inherits the underlying texture. With increasing film thickness, the features progressively smooth due to conformal growth. To capture this effect in the optical modelling, two sample devices are fabricated and characterized: one terminated at the p‐type interface and one at the n‐type interface. Thus, the AZO/nc‐SiO_x_ (p) and nc‐SiO_x_ (n) layers define the first and second optical interfaces, respectively.

**FIGURE 5:**
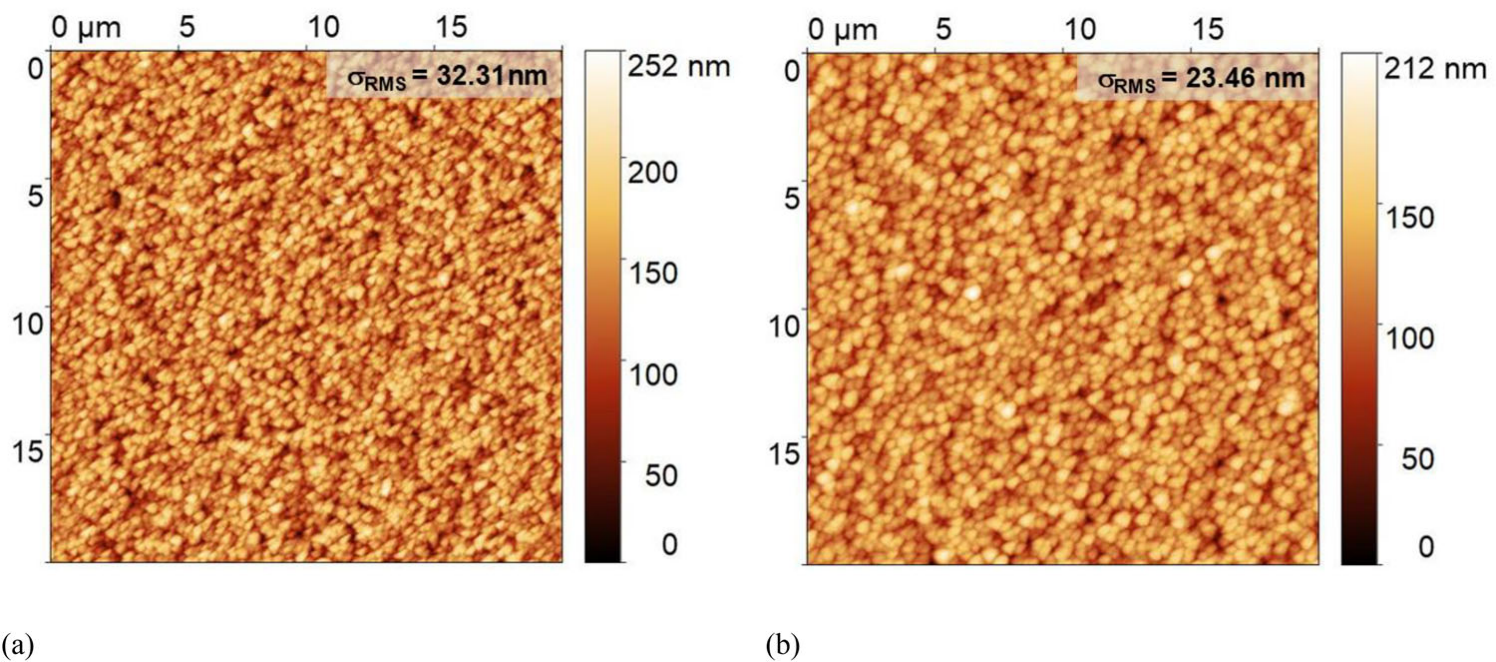
AFM top‐view images of the textured interfaces in the a‐Si:H single‐junction solar cell on Asahi VU‐type glass. (a) Surface after deposition of p‐type nc‐SiO_x_ layer. (b) Surface after subsequent deposition of the intrinsic a‐Si:H absorber.

AFM scans of 20 µm × 20 µm areas (Figure 5) show that deposition of AZO and nc‐SiO_x_ (p) reduces the surface roughness of the original VU‐type substrate (σ_RMS_ = 45 nm, Figure 2) to 32 nm. With deposition of the a‐Si:H absorber, the roughness decreases further to 23 nm, accompanied by a noticeable increase in lateral feature size (Figure 5b). The AFM‐derived height maps of these two interfaces are directly implemented in the ray‐tracing simulations.

**FIGURE 6:**
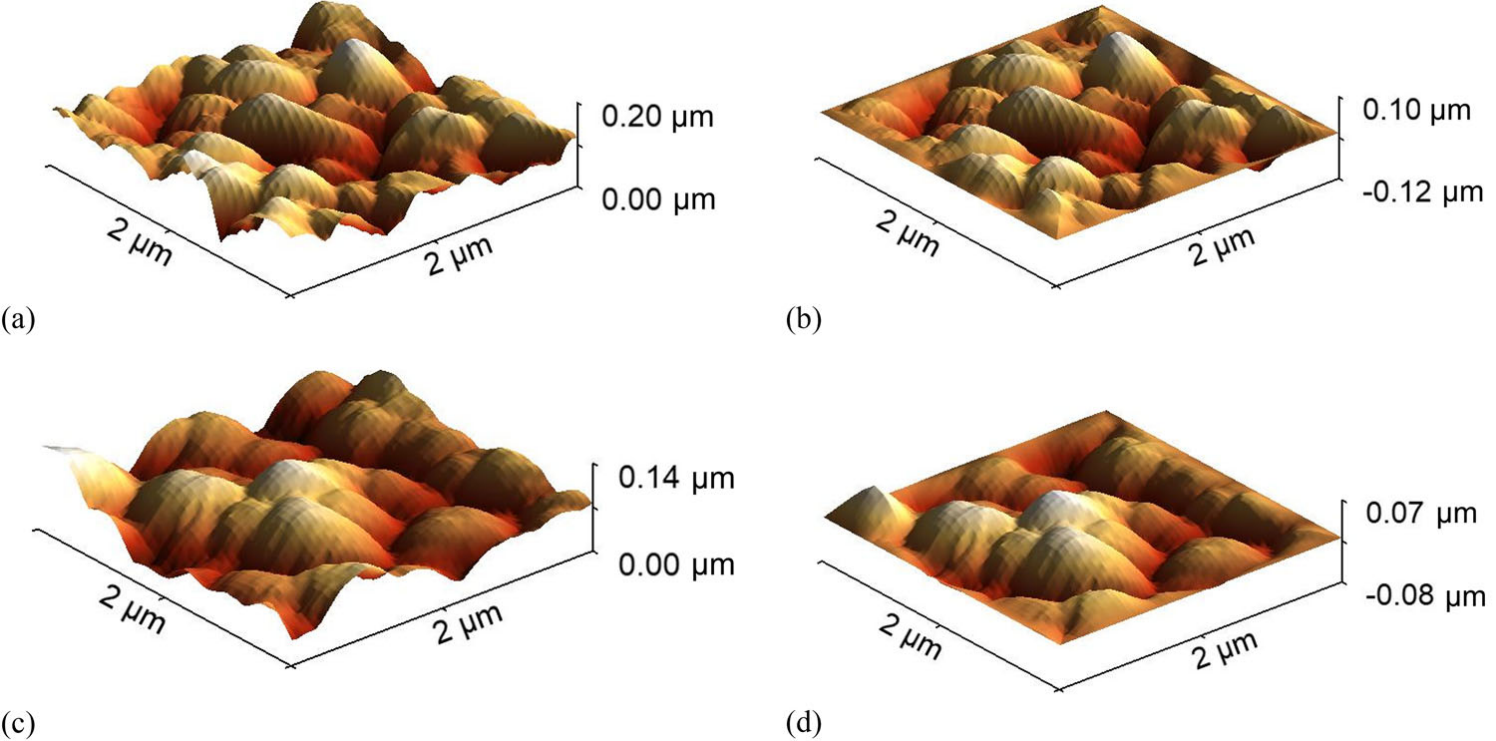
Pre‐processing of AFM‐derived surface morphologies for RCWA simulations of the a‐Si:H solar cell on Asahi VU‐type glass. (a,c) Downscaled 2 µm × 2 µm AFM maps of the interfaces. (b, d) Corresponding morphologies after application of the 3D Tukey window function.

Additional steps are necessary to handle the Asahi VU‐type texture when the optical performance is simulated with the RCWA solver. The AFM maps are first downscaled from 20 µm × 20 µm to 2 µm × 2 µm sections (Figure 6a,c), retaining key morphological features while ensuring computational feasibility. A 3D Tukey window function (see Section 2.1.1) is then applied to suppress edge discontinuities and enable periodic boundary conditions. The pre‐processed height maps are shown in Figure 6b,d are used as RCWA inputs, ensuring numerical stability while preserving the essential characteristics of the experimental textures. The resulting height matrices are discretized on 1001 × 1001 grids to resolve fine surface features. This resolution ensures sufficient refinement in the x, y, and z directions to numerical compatibility with the spatial resolution of the $\vec{E}$ distribution.

#### 3.2.1 Optical Constants

Accurate knowledge of the refractive index and extinction coefficient of all layers in the device stack is essential for reliable optical modelling, regardless of the employed optical model. The FTO‐coated Asahi VU‐type glass, serving as the textured front contact, plays a critical role in light coupling and scattering. Its optical properties depend on deposition method, thickness, and surface morphology [35, 36, 37], and wavelength‐resolved n and k data are required to quantify reflection losses, parasitic absorption, and overall device performance.

**FIGURE 7:**
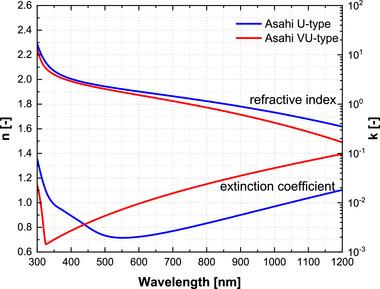
Refractive index (n) and extinction coefficient (k) spectra of FTO‐coated Asahi U‐type and VU‐type glass substrates. The VU‐type data are obtained in this work from ellipsometry fitting, while the U‐type reference data are taken from [38].

Figure 7 compares the complex refractive index of FTO on U‐type and VU‐type substrates. The U‐type results from Sap et al. [38], obtained by fitting reflectance and transmittance spectra with SCOUT software [39], serve as a reference. This work extends the characterization to VU‐type substrate using variable‐angle spectroscopic ellipsometry (SE). The measured amplitude ratio (Ψ) and phase difference (Δ) spectra are fitted in CompleteEASE software [40]. A multilayer optical model is constructed, consisting of a surface SnO_2_ thin film for roughness‐induced modifications, a conductive FTO bulk layer described by Drude‐Lorentz oscillators, and a depth‐dependent conductivity gradient for vertical inhomogeneity in the film.

While the real part (n) remains comparable for the U‐ and VU‐type substrates in the UV–visible range, the extinction coefficient (k) of the VU‐type increases toward the near‐infrared. Because the absorption coefficient is given by α = 4πk/λ, this rise in k directly translates into stronger absorption at long wavelengths [41].

In transparent conducting oxides, such near‐infrared absorption originates from free‐carrier absorption, where conduction‐band electrons absorb low‐energy photons through intra‐band (Drude‐type) transitions. The strength of this process increases with carrier density and decreases with carrier mobility, producing a characteristic long‐wavelength tail in k(λ) [42]. The enhanced k in the NIR region observed for the VU‐type FTO is therefore attributed to its higher free‐carrier absorption, whereas the differences in bandgap energy, film thickness, and surface roughness reported in Table 1 primarily affect visible‐range transmission and scattering. These results therefore, highlight the importance of direct, substrate‐specific optical characterization.

**TABLE 1:** Comparison of optical and morphological parameters of FTO‐coated Asahi U‐type and VU‐type glass substrates. The VU‐type results are obtained in this work: bandgap energy and layer thickness from ellipsometry fitting, and RMS roughness from AFM measurements. The U‐type reference data are taken from [38].

FTO—Asahi	U‐type	VU‐type
Bandgap energy [eV]	4.26	4.05
Layer thickness [nm]	833.4	667.8
RMS roughness [nm]	39.88	45.49

**FIGURE 8:**
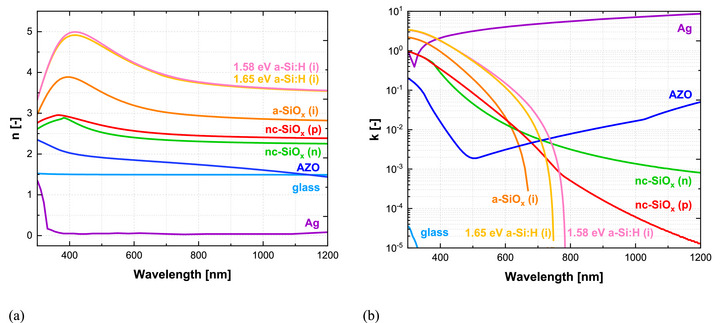
Optical constants of the a‐Si:H single‐junction solar cell on Asahi VU‐type glass. (a) Refractive index (n) spectra and (b) extinction coefficient (k) spectra of all constituent layers, including glass, FTO, AZO, intrinsic a‐SiO_x_, p‐ and n‐type nc‐SiO_x_, and Ag. a‐Si:H films with optical bandgaps of 1.58 eV and 1.65 eV are compared.

The complete n and k spectra for all layers in the a‐Si:H solar cell are shown in Figure 8. For Ag and the glass substrate (excluding the FTO coating), previously measured SE data are used [43, 44]. The glass exhibits a constant n of ∼ 1.5 with negligible absorption. Silver exhibits a refractive index well below 1 and an extinction coefficient above 2 across most of the spectrum, consistent with strong metallic absorption and reflectivity [45].

All remaining materials are deposited and characterized in‐house. Their optical constants are extracted from SE measurements using a Tauc–Lorentz model for inter‐band transitions, with an additional Drude term applied only to layers where free‐carrier absorption becomes relevant in the near‐infrared region.

To improve the short‐circuit current density (J_sc_) of the solar cell, a narrow‐bandgap a‑Si:H material is employed. This material is deposited by plasma‐enhanced chemical vapor deposition (PECVD) under process conditions optimized to lower the bandgap [46, 47]. The resulting film exhibits an optical bandgap of 1.58 eV. Its refractive index peaks near 5.0 at 400 nm and decreases to ∼ 3.5 in the near‐infrared. For comparison, Figure 8 also shows the optical behavior of a wider‐bandgap a‑Si:H material (1.65 eV). The measured data are consistent with previously reported values  [48, 49].

The optical response of the AZO front contact is fitted with a combined Cody–Lorentz and Drude oscillator models, accounting for both inter‐band transitions and free‐carrier absorption. The latter becomes significant in the near‐infrared due to the high conductivity of the material. The extinction coefficient remains low across the visible spectrum, reflecting the transparent nature of AZO. These results are in good agreement with previously reported values [50].

The intrinsic a‐SiO_x_ buffer layer shows a refractive index that peaks at 3.6 around 400 nm and decreases to a stable value of 2.8 in the near‐infrared. The extinction coefficient cuts off at 670–680 nm, corresponding to an optical bandgap of approximately 1.84 eV. These findings are consistent with previous reports [51].

The n‐type and p‐type nc‐SiO_x_ layers exhibit peak refractive indices of approximately 2.8 and 3.0, respectively. Both show moderate absorption in the blue spectral region and negligible extinction beyond 700 nm, reflecting the combined effects of nano‐crystallinity and oxygen incorporation [48, 52, 53].

### 3.3 nc‐Si:H Single‐Junction Solar Cell on Honeycomb Textured Glass

**FIGURE 9:**
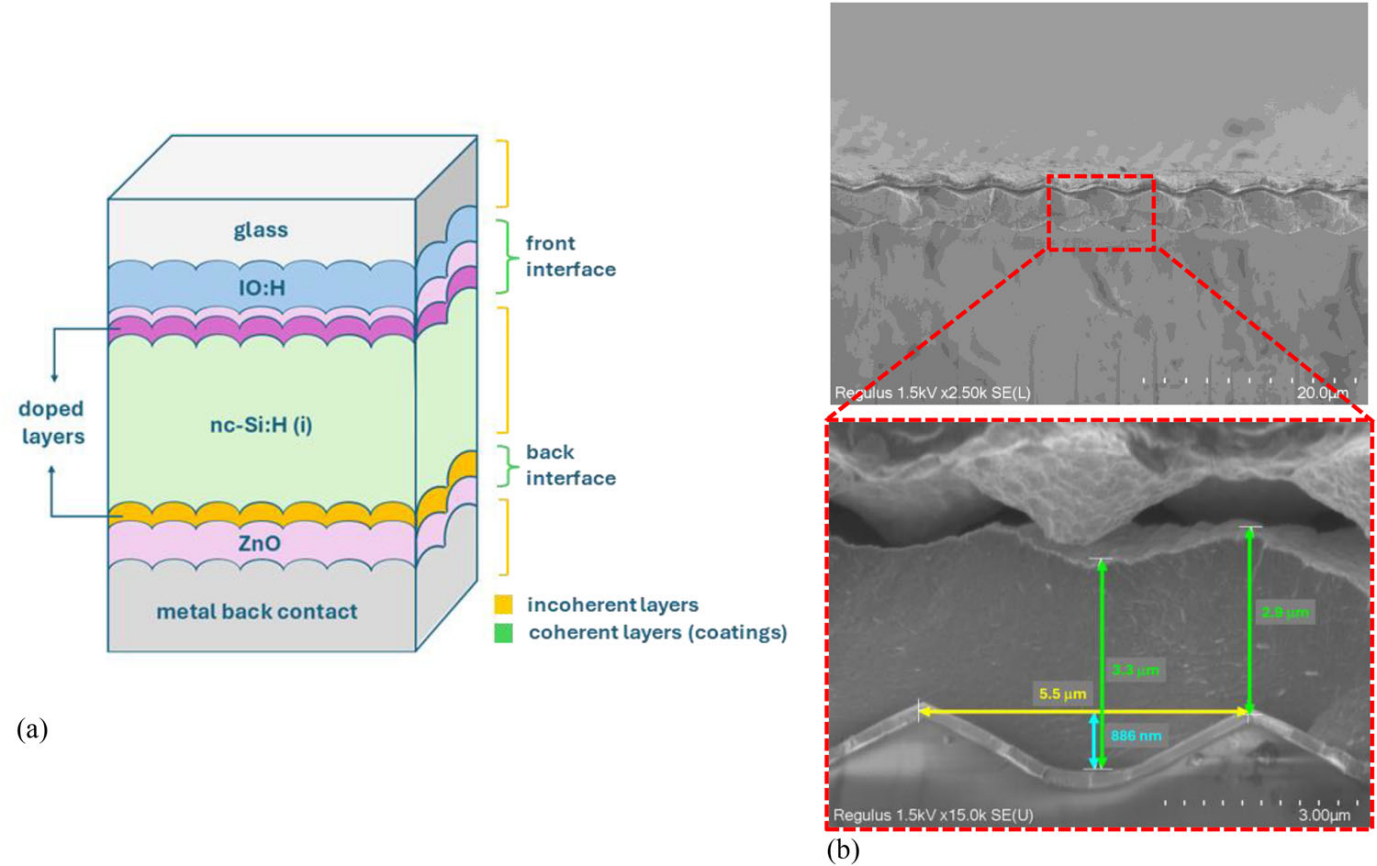
Structure of the nc‐Si:H single‐junction solar cell on honeycomb‐textured glass. (a) Schematic cross‐section of the device stack (not to scale). Layer sequence: glass (0.7 mm)/IOH (135 nm)/ZnO (5 nm)/p‐type nc‐SiO_x_ (10 nm)/intrinsic nc‐SiO_x_ (3 nm)/intrinsic a‐Si:H (3.2 µm)/n‐type nc‐SiO_x_ (20 nm)/ZnO (60 nm)/Ag (300 nm). (b) Cross‐sectional SEM images showing conformal replication of the honeycomb texture throughout the solar cell. The magnified view highlights the preserved honeycomb geometry, with period, depth, and layer thicknesses extracted directly from the SEM.

On the honeycomb‐textured glass substrate, a p‑i‑n nc‑Si:H single‐junction solar cell is fabricated in a superstrate configuration. This architecture exploits the engineered periodic honeycomb pattern to enhance long‐wavelength scattering and improve optical confinement in the thick absorber layer. The device structure is shown in Figure 9, with layer thicknesses not drawn to scale.

The solar cell comprises a 135 nm hydrogenated indium oxide (IO:H) front contact, a 5 nm ZnO buffer layer, and nanocrystalline p‐ and n‐type SiO_x_ layers with thicknesses of 10 nm and 20 nm, respectively. A 3.2 µm intrinsic nc‐Si:H layer serves as the absorber, while the back reflector consists of a 60 nm ZnO layer and a 300 nm Ag contact. As noted in Section 3.2, the back contact is optically simplified for modeling purposes.

A key feature of this architecture is the conformal replication of the honeycomb geometry throughout the entire stack. This is confirmed by cross‐sectional SEM imaging (Figure 9b), which shows that the texture is faithfully preserved across all deposited layers. From the same SEM, the honeycomb period, depth, and individual layer thicknesses are determined, confirming both structural integrity and fabrication accuracy. The apparent gap visible between the back contact and the underlying deposition stack arises from mechanical damage introduced during sample cleaving for cross‐sectional imaging and is not present in the actual device.

**FIGURE 10:**
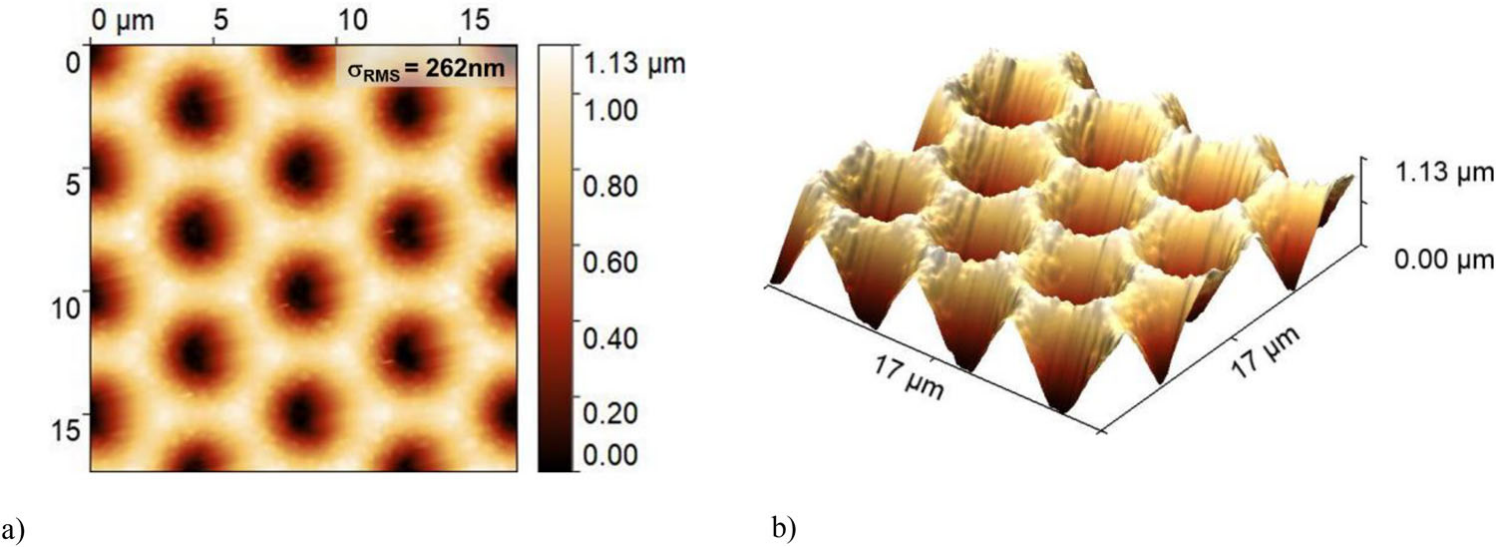
AFM scans of the absorber surface in the nc‐Si:H single‐junction solar cell fabricated on honeycomb‐textured glass: (a) 2D top view and (b) 3D morphology.

Optically, two textured interfaces are defined in the model, where the optical solvers (ray tracing or RCWA) are applied to resolve the angular intensity distribution of scattered light. These correspond to the front and rear sides of the intrinsic nc‐Si:H absorber, where texturing governs light coupling and redistribution within the device. The first interface, at the front of the absorber, comprises the IO:H, ZnO, and p‐type SiO_x_ layers, located between the glass substrate and the intrinsic nc‐Si:H. Both the glass and nc‐Si:H are modeled as incoherent layers. This textured interface follows the honeycomb geometry shown in Figure 3 (Section 3.1). The second interface, at the rear of the absorber, includes the n‐type SiO_x_ and ZnO layers at the nc‐Si:H/Ag boundary. AFM scans of the absorber surface (Figure 10) reveal that the honeycomb texture is preserved after nc‐Si:H deposition. Fine‐scale nanofeatures developed during nc‑Si:H growth, without significantly affecting the overall macro‐scale texture. The σ_RMS_ remains approximately 262 nm, indicating that the macro‐scale roughness profile is maintained.

As with the Asahi VU‐type texture (Section 3.2), implementing the honeycomb texturing in the RCWA model requires defining a finite, periodic unit cell compatible with the boundary conditions. In this case, the full geometry of a single honeycomb unit can be effectively captured within a 6 µm × 6 µm section, which sufficiently represents the periodicity and structural features of the texture. To enforce RCWA boundary conditions, the unit‐cell edges are gradually tapered to zero height, avoiding abrupt discontinuities and ensuring numerical stability during Fourier decomposition. The resulting height matrices are discretized on 1001 × 1001 grids to resolve surface features. This resolution ensures sufficient refinement in the x, y, and z directions to numerical compatibility with the spatial resolution of the $\vec{E}$ distribution.

**FIGURE 11:**
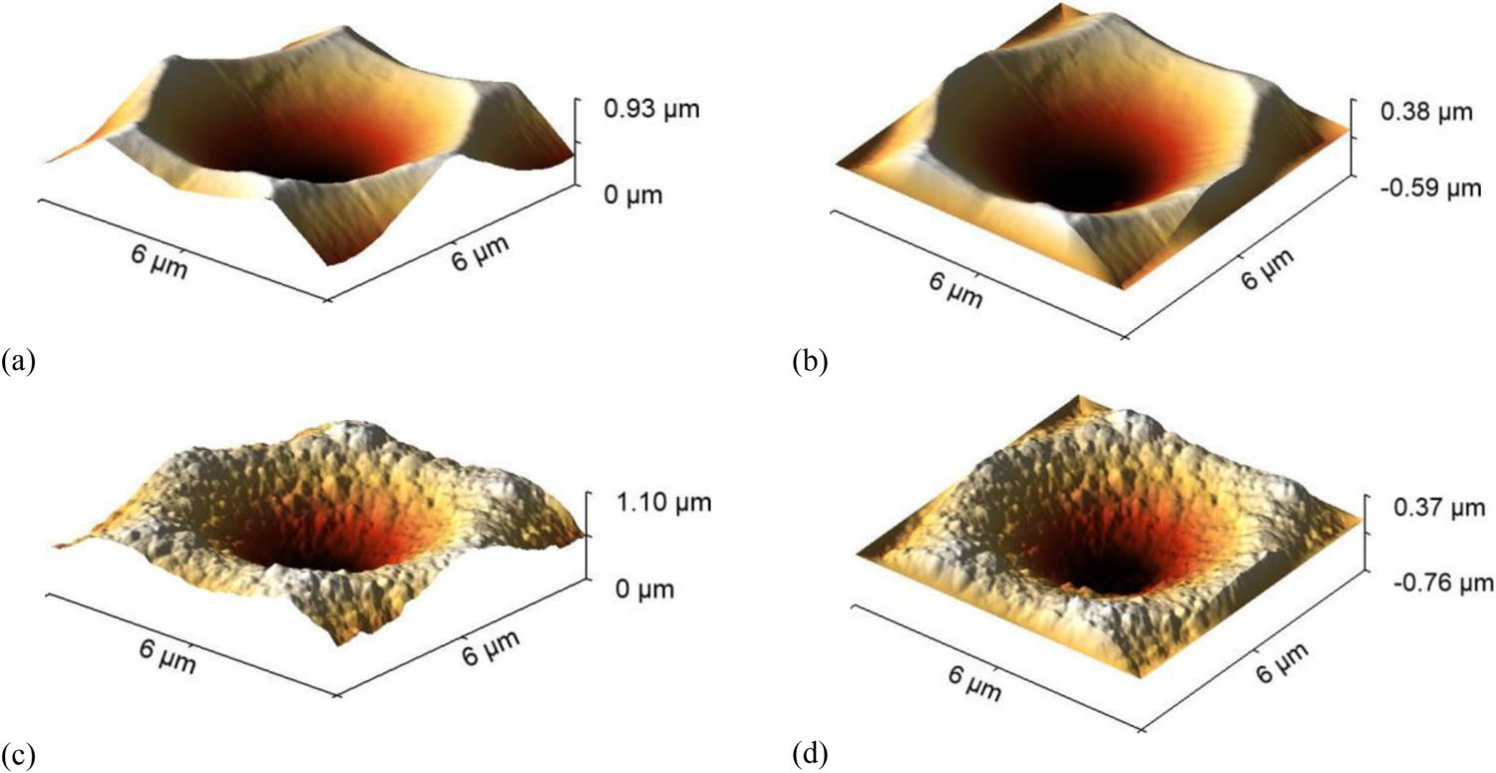
Preprocessing of AFM‐derived honeycomb morphologies for RCWA simulations. (a) 6 µm × 6 µm unit cell of the front interface and (b) same unit cell after application of the 3D Tukey window function. (c) 6 µm × 6 µm unit cell of the rear interface and (d) same unit cell after Tukey window preprocessing.

Figure 11a shows a 6 µm × 6 µm honeycomb unit cell extracted from the AFM morphology of the front interface (Figure 3, Section 3.1), and Figure 11c shows the corresponding unit cell of the rear interface (Figure 10). Figure 11b,d display the same structures after application of the 3D Tukey window function. This preprocessing step enables accurate representation of the honeycomb morphology in RCWA while maintaining computational feasibility and minimizing boundary artifacts.

#### 3.3.1 Optical Constants

**FIGURE 12:**
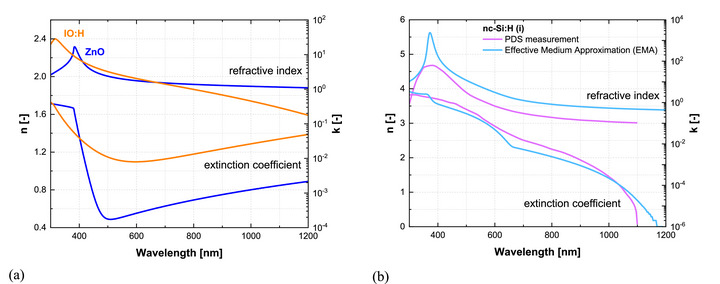
Refractive index (n) and extinction coefficient (k) spectra of: (a) hydrogenated indium oxide (IO:H) and zinc oxide (ZnO); (b) intrinsic nc‐Si:H, with optical constants obtained from photothermal deflection spectroscopy (PDS) [15] and from effective medium approximation (EMA) fitting of ellipsometry data in this work.

The optical constants of the TCO layers at the front electrode and of the intrinsic nc‐Si:H absorber are shown in Figure 12. The optical properties of the remaining layers in the device, the glass substrate, the p‐type and n‐type SiO_x_ layers, and the Ag back contact, are provided in Figure 8 and discussed in Section 3.2.1.

Figure 12a presents the refractive index and extinction coefficient spectra of the optimized TCO stack, consisting of hydrogenated indium oxide (IO:H) and zinc oxide (ZnO). The optical constants are derived from spectroscopic ellipsometry and fitted using combined Cody–Lorentz and Drude oscillator models. This approach captures refractive index dispersion, ultraviolet absorption behavior, and free carrier absorption in the near‐infrared. The TCO layers are deposited under optimized process conditions to balance optical transparency and electrical conductivity, ensuring high transmission while maintaining sufficient carrier transport properties [54].

Optically, IO:H exhibits a peak refractive index of 2.4 at 320 nm, while ZnO peaks at 2.3 near 380 nm. The optical bandgaps are approximately 3.7 eV for IO:H and 3.0 eV for ZnO. IO:H shows stronger free‐carrier absorption in the near‐infrared, which reduces the transmitted light reaching the absorber at longer wavelengths, whereas ZnO maintains higher transmittance in this region. Careful control of free‐carrier absorption in IO:H is therefore essential to minimize parasitic losses and sustain high photocurrent generation in devices where absorption extends beyond 1 µm.

Accurate nc‐Si:H optical constants are also critical for realistic device simulation but remain challenging due to the material's heterogenous microstructure. Figure 12b compares refractive index and extinction coefficient spectra of the intrinsic nc‐Si:H material obtained by photothermal deflection spectroscopy (PDS) [55] and by effective medium approximation (EMA) [56, 57, 58].

In the PDS method, as described in previous studies [15], parasitic absorption contributions such as defect‐related absorption are subtracted from the raw data to approximate a more realistic extinction coefficient. Additional corrections account for interference effects arising from film inhomogeneity along the growth direction, which may influence the representativeness of the derived optical constants.

In this work, the optical constants are extracted from spectroscopic ellipsometry of a 250 nm nc‐Si:H film deposited on Corning glass. The dielectric function is modeled using a Bruggeman EMA, treating the film as a mixture of crystalline and amorphous silicon phases. The optical constants of the constituent materials are taken from the J.A. Woollam and Palik datasets [40, 59], which originate from spectroscopic‐ellipsometry measurements analyzed using Kramers–Kronig–consistent dispersion models. The EMA is then applied to the effective complex dielectric function, where both the real and imaginary components are calculated self‐consistently. Because the Bruggeman formalism operates directly on this complex function, the resulting effective refractive index and extinction coefficient preserve the Kramers–Kronig relationship.

The method assumes bulk‐like polarizability of the constituents and structural homogeneity on the wavelength scale, conditions that are satisfied for the optimized material developed here. Previous studies indicate that a crystalline volume fraction of 60–65 % yields optimal properties for solar cells, which is consistently achieved in this work through precise control of plasma conditions during growth [60].

The EMA approach provides smooth spectral trends and avoids artifacts associated with PDS. The derived constants include an optical bandgap close to 1.12 eV and reliably capture the dielectric response of the material, offering a solid basis for optical modeling of the nc‐Si:H solar cell.

### 3.4 Optical Models Summary

Table 2 summarizes the key differences between RCWA and ray‐optics solvers. RCWA requires periodic boundary conditions; thus, measured textures are downscaled and tapered at the unit‐cell edges using a Tukey window to enforce continuity. Ray optics does not impose such constraints and can directly incorporate the measured morphology.

**TABLE 2:** Key differences between rigorous coupled‐wave analysis (RCWA) and ray optics solvers in the optical modeling of textured thin‐film silicon solar cells.

Aspect	RCWA	Ray optics
Boundary conditions	Texture approximation required	Not applicable
Texture	Downscaled AFM maps + Tukey window (tapered edges to zero)	AFM maps
Coherent layers	Front and back interfaces	Front and back interfaces
Optical constants	Experimentally characterized	Experimentally characterized
Computation time	∼ 1 week	< 30 min

In single‐junction architectures, the absorber layer is treated incoherently. The thin layers between the glass substrate and the absorber define the front interface, while those between the absorber and the metallic back contact define the back interface of the optical design. At each defined interface, the corresponding AFM morphology is either preserved or reshaped depending on the solver's input requirements. Both methods rely on experimentally determined optical constants of the device materials. The most pronounced difference lies in computational demand: RCWA typically requires about 1 week for device‐scale runs, whereas ray optics simulation completes within 30 min. Further details on computation time and resolution are provided in Section 4.1 and Section 4.2 for the a‐Si:H and nc‐Si:H single‐junction devices, respectively.

## 4 Simulation Output: Single‐junction Solar Cells Validation

This section presents the simulated optical performance of thin‐film Si‐based single‐junction solar cells using GenPro4. Optical predictions from ray optics and rigorous coupled‐wave analysis are compared with experimental measurements of external quantum efficiency (EQE) and total front reflection losses (1‐R) from in‐house fabricated devices. Section 4.1 focuses on the a‐Si:H single‐junction solar cell on Asahi VU‐type glass, while Section 4.2 examines the nc‐Si:H single‐junction solar cell on honeycomb‐textured glass.

### 4.1 Optical Performance of a‐Si:H Solar Cell

**FIGURE 13:**
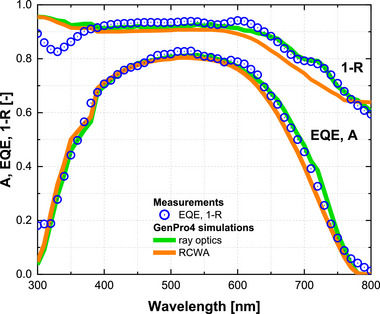
Optical performance of the a‐Si:H single‐junction solar cell on Asahi VU‐type glass. Blue dots represent experimental measurements of external quantum efficiency (EQE) and total front reflection losses (1‐R). Green lines show simulated absorptance (A) in the a‐Si:H absorber and 1‐R obtained with the ray optics model, while orange lines show the corresponding results from RCWA simulations.

Figure 13 compares the optical performance predicted by ray optics and RCWA with experimental measurements for the a‐Si:H single‐junction solar cell on Asahi VU‐type glass. Both models are applied to the same device structure (Section 3.2), using a wavelength interval of 10 nm to ensure consistency. RCWA simulations are run with numerical settings optimized for convergence: the device is discretized into 125 sublayers, the local electric field is resolved on 25 points in the x, y, and z directions, and 17 Fourier modes are used, which have been shown to provide sufficient accuracy for thin‐film solar cell structures [61, 62]. Under these settings, RCWA simulations require approximately 1 week of computation, whereas the ray optics model completes within 30 min.

The ray optics model shows close agreement with experiment: the simulated a‐Si:H absorptance matches the measured EQE, and the simulated 1‐R reproduces the measured reflection losses across the full spectral range. In contrast, RCWA simulation shows noticeable deviations, particularly in the near‐infrared, where absorption is underestimated and reflection losses are overestimated. A distinct dip between 300–350 nm in the measured reflection profile may originate from interference effects at the glass/FTO interface, which are not fully captured by either model.

Table 3 summarizes the short‐circuit current density (J_sc_) measured from the EQE and the implied photocurrent density predicted by the models. Assuming every absorbed photon in the a‐Si:H absorber contributes to charge collection (i.e., negligible electrical losses), the J_sc_ extracted from EQE agrees closely with the J_ph_ from the ray optics model, indicating its predictive accuracy. The photocurrent estimated by the RCWA model is noticeably lower than the measured J_sc_, reflecting limitations of this approach under the current simulation conditions.

**TABLE 3:** Short‐circuit current density (J_sc_) measured from EQE, with an experimental uncertainty of ± 0.1 mA/cm^2^, and implied photocurrent density (J_ph_) predicted by ray optics and RCWA for the a‐Si:H single‐junction solar cell on Asahi VU‐type glass.

Method	J_ph_ or J_sc, EQE_ [mA/cm^2^]
RCWA	15.62
Ray‐optics	16.08
EQE measurement	16.01

A key limitation arises from the Asahi VU‐type substrate morphology: its non‐periodic nanofeatures are not fully compatible with the inherently periodic RCWA framework, which can lead to inaccuracies in scattering and angular redistribution. Despite its theoretical rigor, RCWA is therefore less suited for this case, whereas the simpler and faster ray optics model provides a more reliable description of the optical behavior of the a‐Si:H solar cell.

Over the spectral range of 300–800 nm, the root mean squared error (defined in Section 2, Equation 2) amounts to 3.6% for the ray optics and 3.3% for RCWA when comparing simulated absorptance with measured EQE. These values confirm that both models reproduce EQE with good accuracy, with RCWA performing slightly better on average. For 1‐R, the deviations are 3.7% for ray optics and 4.7% for RCWA, consistent with the trends in Figure 13, where the ray optics model better captures the experimental reflectance across the spectrum. Together with the J_sc_ analysis in Table 3, these results indicate that, while both approaches are quantitatively reliable, the ray optics model achieves comparable or better agreement with experiment while offering substantially greater computational efficiency for simulating the a‐Si:H solar cell on Asahi VU‐type glass.

#### 4.1.1 Scattering Matrices

**FIGURE 14:**
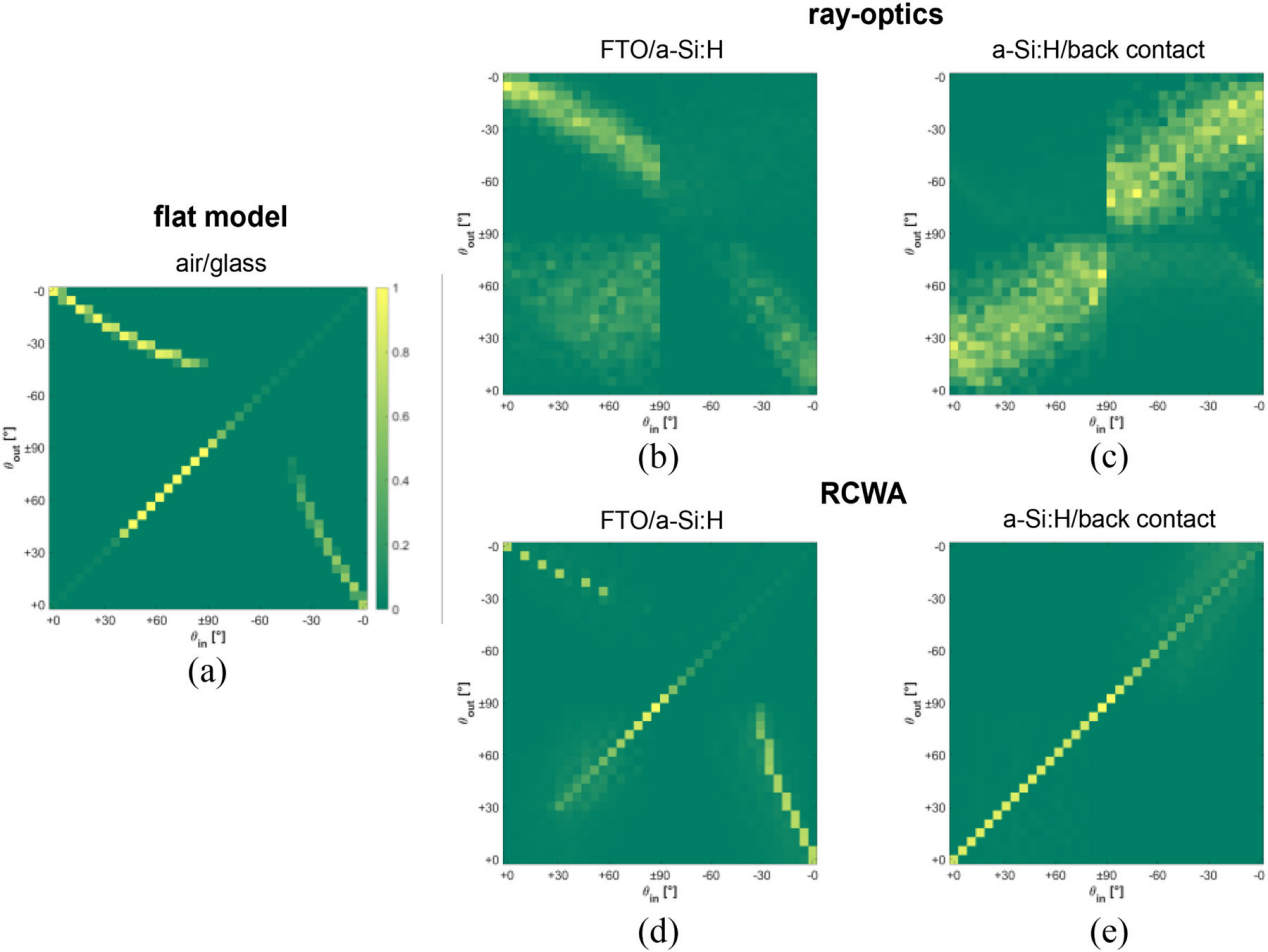
Angular scattering matrices of the a‐Si:H single‐junction solar cell on Asahi VU‐type glass. (a) Flat air/glass interface. (b, c) glass–absorber and absorber/back‐contact interfaces from ray optics. (d, e) Corresponding interfaces from RCWA. The matrices are plotted as 2D maps, where the x‐axis represents the incident angle (θ_in_) and the y‐axis the scattered angle (θ_out_), both defined over the range ± 90° relative to the surface normal.

Figure 14 shows the angular scattering matrices calculated at different interfaces of the a‐Si:H solar cell using ray optics and RCWA. In GenPro4, scattering matrices quantify the probability of light being reflected or transmitted between angular intervals on both sides of an interface [24]. Each is represented as a 2D map, with columns corresponding to incoming angles and rows to outgoing angles, covering the full ± 90° range relative to the surface normal.

At the flat air/glass interface (Figure 14a), reflection is purely specular and transmission follows Snell's law due to the refractive index contrast. Figure 14b,d shows the scattering matrices of the FTO‐coated Asahi glass/a‐Si:H absorber interface calculated by ray optics and RCWA, respectively. Figure 14c,e provide the corresponding results for the absorber/back‐contact interface.

Ray optics (Figure 14b,c) produces broad angular redistribution, with significant scattering into oblique angles, consistent with the diffuse behavior of the random VU‐type texture. RCWA (Figure 14d,e), in contrast, confines energy to the specular direction and a few discrete diffraction orders, reflecting its intrinsic limitation when applied to non‐periodic morphologies.

These differences directly influence absorption and photocurrent generation. By capturing diffuse angular redistribution, ray optics reproduces the light‐trapping effects of the Asahi VU‐type glass and aligns with experimental absorption trends. RCWA underestimates diffuse scattering and, as a result, fails to fully describe long‐wavelength confinement in this device architecture.

### 4.2 Optical Performance of nc‐Si:H Solar Cell

**FIGURE 15:**
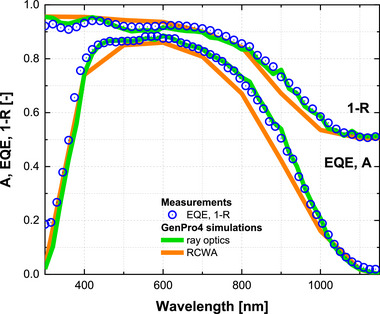
Optical performance of the nc‐Si:H single‐junction solar cell on honeycomb‐textured glass. Blue dots represent experimental measurements of external quantum efficiency (EQE) and total front reflection losses (1‐R). Green lines show simulated absorptance (A) in the nc‐Si:H absorber and 1‐R obtained with the ray optics model, while orange lines show the corresponding results from RCWA simulations.

Figure 15 compares the optical performance predicted by ray optics and RCWA with experimental measurements for the nc‐Si:H single‐junction solar cell on honeycomb‐textured glass. RCWA simulations are run with numerical settings optimized for convergence: the device structure is discretized into 100 sublayers, the local electric field is resolved with 50 nm spacing in the x‐y plane and 10 nm along z direction, and 17 Fourier modes are used. To limit computation to about 1 week, the spectral interval is set to 100 nm. The ray optics model employs a finer 20 nm interval, consistent with the measurement sampling, at a negligible computational cost.

The ray optics model shows very good agreement with experiment, reproducing both the absorptance in the nc‐Si:H layer and the 1‐R losses across the full spectral range. RCWA, by comparison, systematically underestimates absorption and overestimates reflection, with the largest deviations between 800 and 1100 nm. Moreover, RCWA does not capture the interference feature observed between 400 and 500 nm, originating from the TCO front contact.

Table 4 summarizes the J_sc_ extracted from EQE and the J_ph_ predicted by the models, under the assumption that each absorbed photon generates a collected carrier. The J_sc_ obtained from EQE shows excellent agreement with J_ph_ from the ray optics model, demonstrating its high predictive accuracy. The RCWA model yields a significantly lower photocurrent, reflecting its limited ability to describe light trapping and scattering in this architecture under the chosen input settings.

**TABLE 4:** Short‐circuit current density (J_sc_) measured from EQE, with an experimental uncertainty of ± 0.1 mA/cm^2^, and implied photocurrent density (J_ph_) predicted by ray optics and RCWA for the nc‐Si:H single‐junction solar cell on honeycomb‐textured glass.

Method	J_ph_ or J_sc, EQE_ [mA/cm^2^]
RCWA	27.9
Ray‐optics	28.9
EQE measurement	28.9

Over the 300–1150 nm range, the absorptance predicted by ray optics deviates on average by 4.0% from the measured EQE, while RCWA shows a larger deviation of 5.2%. For 1‐R, the ray optics model again provides the closer match with an average deviation of 1.7%, compared to 3.2% for RCWA. The larger deviations observed for RCWA should not be interpreted as fundamental shortcomings of the method, but rather as a consequence of limited parameter optimization in this study. With further refinement of numerical settings and input preprocessing, the accuracy of RCWA could improve. Nevertheless, for the present case, ray optics provides a more accurate, reliable, and markedly faster description of the optical performance of the nc‐Si:H solar cell on honeycomb‐textured glass.

#### 4.2.1 Scattering Matrices

**FIGURE 16:**
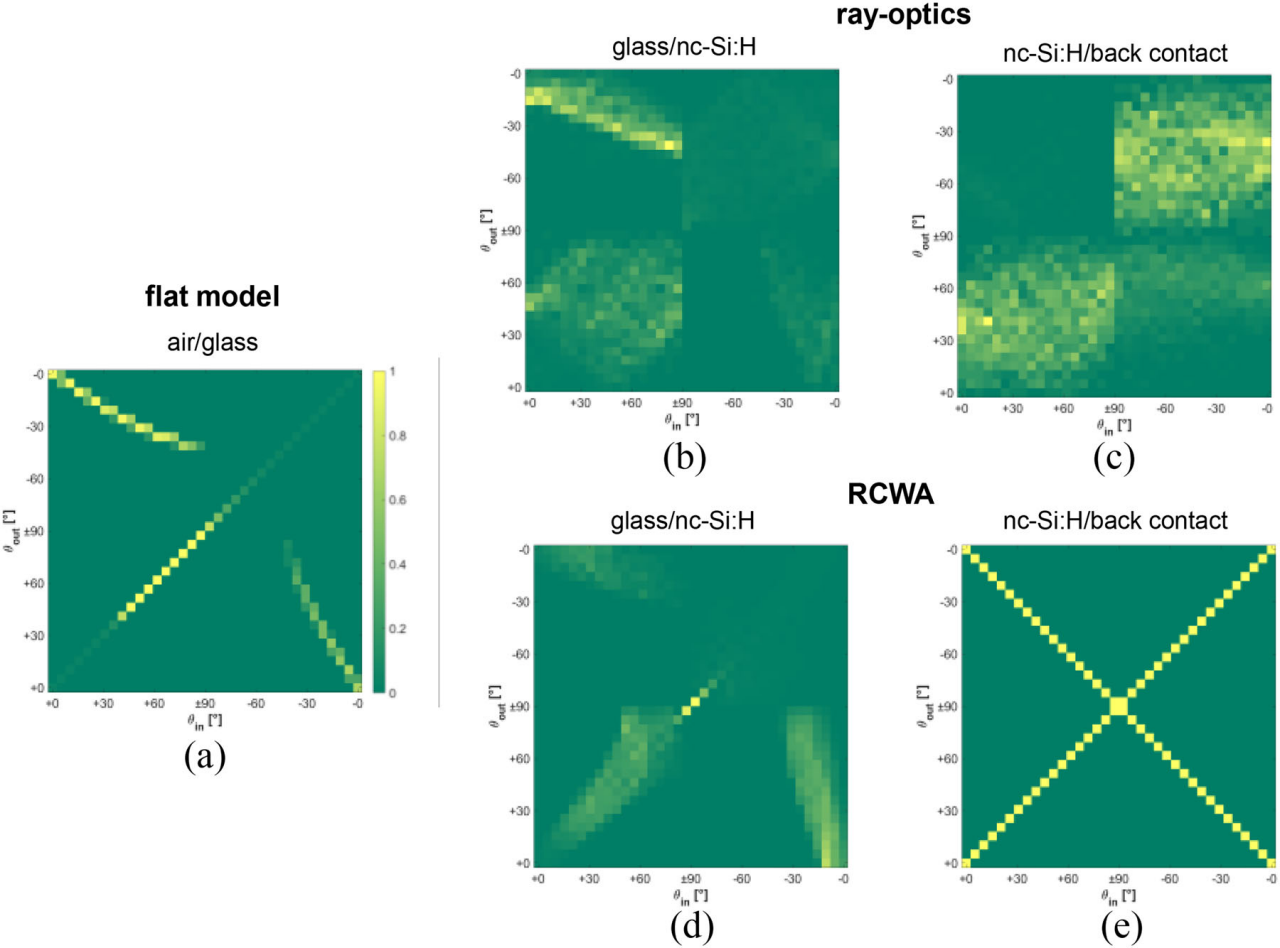
Angular scattering matrices of the nc‐Si:H single‐junction solar cell on honeycomb‐textured glass. (a) Flat air/glass interface. (b, c) glass–absorber and absorber/back‐contact interfaces from ray optics. (d, e) Corresponding interfaces from RCWA. The matrices are plotted as 2D maps, where the x‐axis represents the incident angle (θ_in_) and the y‐axis the scattered angle (θ_out_), both defined over the range ± 90° relative to the surface normal.

Figure 16 compares the angular redistribution of light in the nc‐Si:H single‐junction solar cell fabricated on honeycomb‐textured glass. Figure 16a presents the flat air/glass interface, which exhibits purely specular reflection and refraction. Figure 16b,d shows the scattering matrices at the front textured interface between glass and nc‐Si:H absorber, calculated using ray optics and RCWA, respectively. Figure 16c,e presents the corresponding results at the rear nc‐Si:H/back contact interface.

As shown in Figure 16b, ray optics predicts a broad and diffuse angular redistribution at the glass/nc‐Si:H interface, with significant energy directed into oblique angles. This angular spread is wider than in the Asahi VU‐type case (Figure 14, Section 4.1.1), reflecting the highly regular and deeper honeycomb morphology that enhances long‐wavelength light trapping. The RCWA result for the same interface (Figure 16d) indicates a slightly broader distribution compared to the Asahi VU‐type substrate, but remains confined to discrete diffraction orders, underestimating diffuse scattering.

At the rear nc‐Si:H/back contact interface, ray optics (Figure 16c) shows strong angular diffusion and redirection into large angles, thereby enhancing internal reflection and optical path length. In contrast, the RCWA result (Figure 16e) exhibits a pronounced X‐shaped pattern, likely originating from limited spatial resolution in capturing the complex back texture. These results underscore the effectiveness of ray optics in reproducing realistic diffuse scattering at both front and rear interfaces of the periodic, microtextured nc‐Si:H device.

## 5 Simulation Output: a‐Si:H/nc‐Si:H Tandem Solar Cell

The optical modeling is validated on single‐junction devices, namely an a‐Si:H solar cell on a randomly nanotextured Asahi VU‐type substrate (Section 4.1) and an nc‐Si:H solar cell on a periodically microtextured honeycomb substrate (Section 4.2). Two complementary solvers are employed: a simplified refractive‐regime approach based on ray tracing and a full wave‐optical solver based on RCWA. While RCWA, grounded in rigorous electromagnetic theory, faces challenges in handling experimentally realistic inputs and required significantly higher computational effort, the ray optics model, when combined with accurate optical constants and realistic interface morphologies, simultaneously reproduces both absorber‐layer absorptance and total front reflection losses. These findings establish ray tracing as a robust and efficient predictive tool for thin‐film Si devices.

Building on this validation, the investigation is extended to a‐Si:H/nc‐Si:H tandem solar cell fabricated on honeycomb‐textured glass. Tandem configurations leverage the complementary absorption ranges of wide‐bandgap a‐Si:H (1.65 eV) in the top cell (ToC) and narrow‐bandgap nc‐Si:H (1.12 eV) in the bottom cell (BoC), enabling improved utilization of the solar spectrum. Accurate prediction of the optical response is particularly critical in this case, as both current matching and light‐trapping efficiency determine device performance. Accordingly, ray optics simulations, validated as reliable and computationally efficient, are employed here in combination with detailed material characterization and morphological inputs to assess the optical performance of the tandem structure. In practice, the simulations are conducted with wavelength resolution of 10 nm, keeping the total runtime below 30 min.

**FIGURE 17:**
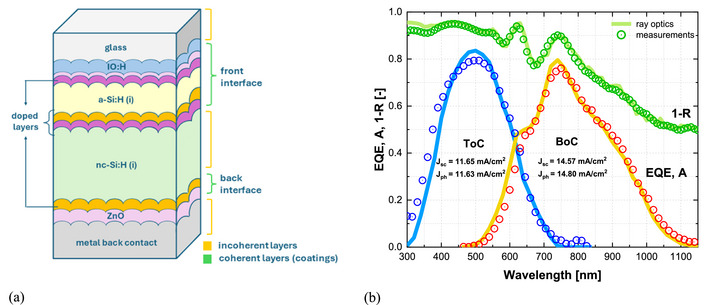
a‐Si:H/nc‐Si:H tandem solar cell on honeycomb‐textured glass. (a) Schematic device structure (layers not to scale). (b) Optical performance: measured external quantum efficiency (EQE) of the a‐Si:H top cell (ToC, blue dots) and nc‐Si:H bottom cell (BoC, red dots), with corresponding simulated absorptance (A) shown as blue and yellow lines. Total front reflection losses (1 − R) are shown as green dots (measured) and a green line (simulated). Short‐circuit current density (J_sc_) measured from EQE, with an experimental uncertainty of ± 0.1 mA/cm^2^, and implied photocurrent density (J_ph_) predicted by ray optics are also reported for ToC and BoC.

The device structure is schematically shown in Figure 17a. For visual clarity, the layers are not drawn to scale. The transparent front electrode consists of a 130 nm IO:H layer and a 5 nm ZnO buffer, providing both optical transparency and electrical conductivity. The ToC is a p–i–n junction composed of a 20 nm p‐type SiOx layer, a 3 nm intrinsic a‐SiOx buffer, a 300 nm a‐Si:H absorber, and a 50 nm n‐type SiOx layer. The BoC is likewise a p–i–n junction, incorporating a 3 µm nc‐Si:H absorber sandwiched between 20 nm p‐type and 50 nm n‐type SiOx layers. The rear stack consists of an 80 nm ZnO back reflector and a 300 nm Ag back contact. Optical constants for all layers are provided in Figure 8 (Section 3.2) and 12 (Section 3.3).

In the optical model, the TCO front contact and the entire a‐Si:H top cell are treated coherently as an assumed simplification, since their sub‐micrometer thicknesses are within the coherence length of light and dominated by interference effects. The thick nc‐Si:H absorber in the bottom cell is instead treated incoherently due to its micrometer‐scale thickness and strong internal scattering, which randomize phase and suppress coherent interference. The front textured interface in the model is defined by all layers between the glass substrate and the intrinsic nc‐Si:H absorber. The rear textured interface consists of the n‐type SiO_x_ of the bottom cell together with the ZnO/Ag back reflector. The honeycomb texture is implemented at both the front and rear interfaces, following the same assumptions applied to the nc‐Si:H single‐junction cell (Section 4.2), where conformal growth preserves the textured morphology throughout the device stack.

Figure 17b compares the measured reflection losses (1‐R) and EQE spectra with the modeled optical response. The agreement is very good across the full spectral range, with both the spectral shape and interference fringes between 550–800 nm consistently reproduced. This demonstrates that the combination of ray optics with coherent treatment of thin layers captures both scattering and interference effects. For the ToC, the simulated absorptance is slightly lower than the EQE between 300–450 nm, suggesting a modest overestimation of parasitic absorption in the supporting layers, while the EQE peak is lower than predicted, likely due to measurement‐related uncertainties. For the BoC, the EQE response closely follows the modeled absorptance across the spectrum, confirming accurate transmission through the ToC and absorption in the nc‐Si:H layer. The comparison of short‐circuit current densities with implied photocurrent densities further supports this consistency, showing that performance is limited by the ToC.

The quantitative comparison between modeled and measured spectra for the a‐Si:H/nc‐Si:H tandem solar cell is performed using the RMSE metric defined in Section 2, Equation 2. Over the 300–800 nm range, the absorptance predicted for the a‐Si:H top cell deviates on average by 6.1% from the measured external quantum efficiency, while in the 450–1150 nm range the nc‐Si:H bottom cell shows a deviation of 3.6%. For the total front reflection losses over 300–1150 nm, the deviation between simulation and experiment is 1.7%. These results confirm that the ray optics model, when complemented by coherent treatment of thin layers, accurate optical constants, and realistic interface morphologies, provides a reliable and quantitatively accurate description of the optical performance of the a‐Si:H/nc‐Si:H tandem device on honeycomb‐textured glass.

## 6 Conclusion

The concept of thin‐film silicon solar cells was adopted to investigate how accurately the optical performance of devices on multiscale‐textured substrates can be predicted. Two representative architectures were considered and fabricated in‐house: an a‐Si:H cell on randomly nanotextured Asahi glass and an nc‐Si:H cell on a novel micro‐periodic honeycomb‐textured glass. In both cases, the analysis extended beyond conventional optical device design, incorporating thorough characterization of optical constants and realistic morphologies of the main interfaces directly into the simulations.

For the first time, a direct comparison was made between a simplified ray optics model, operating in the refractive regime, and rigorous coupled‐wave analysis (RCWA). RCWA captured electromagnetic scattering mechanisms in full detail, but its use was limited by periodic boundary requirements and extremely high computational demand. Ray optics, by contrast, directly incorporated realistic morphologies without such constraints. When supplied with experimentally determined inputs, it reproduced absorptance and reflection spectra with accuracy better than RCWA, while reducing computation time from 1 week to less than 30 min.

By benchmarking against measured external quantum efficiency (EQE) and total front reflection losses (1‐R) of single‐junction a‐Si:H and nc‐Si:H solar cells, the predictive accuracy of both methods was quantified using the root mean square error (RMSE) metric. Across all cases, the ray optics model achieved deviations comparable to or smaller than RCWA (typically 2%–6%), while maintaining superior computational efficiency. Building on this validation, the ray optics model was applied to an a‐Si:H/nc‐Si:H tandem solar cell on honeycomb‐textured glass, representing a more challenging optical system. The simulations reproduced the measured optical response with deviations within 6%. Importantly, the photocurrent densities (J_ph_) and short‐circuit currents (J_sc_) were predicted with a current mismatch below 0.2 mA/cm^2^, indicating both quantitative accuracy and robustness of the method.

These findings provide the first direct demonstration that ray optics, when combined with accurate optical constants and detailed morphological input, can reliably capture not only absorption and reflection but also the angular redistribution of light induced by multiscale‐textured interfaces. This unique validation establishes ray tracing as a powerful and computationally efficient predictive tool for thin‐film silicon photovoltaics. Beyond silicon, this methodology is broadly transferable to other thin‐film technologies, including perovskites, where multiscale light management is central to next‐generation photovoltaic design.

## Funding

Bilateral collaboration between HyET Solar Netherlands B.V. and Delft University of Technology.

## Conflicts of Interest

The authors declare no conflict of interest.

## Data Availability

The data that support the findings of this study are available from the corresponding author upon reasonable request.
